# Identification of early changes in specific symptoms that predict longer-term response to atypical antipsychotics in the treatment of patients with schizophrenia

**DOI:** 10.1186/1471-244X-11-23

**Published:** 2011-02-09

**Authors:** Stephen J Ruberg, Lei Chen, Virginia Stauffer, Haya Ascher-Svanum, Sara Kollack-Walker, Robert R Conley, John Kane, Bruce J Kinon

**Affiliations:** 1 Eli Lilly and Company, Indianapolis, IN, USA; 2 Lilly USA, LLC, Indianapolis, IN, USA; 3 Zucker Hillside Hospital, Glen Oaks, NY, USA

## Abstract

**Background:**

To identify a simple decision tree using early symptom change to predict response to atypical antipsychotic therapy in patients with (Diagnostic and Statistical Manual, Fourth Edition, Text Revised) chronic schizophrenia.

**Methods:**

Data were pooled from moderately to severely ill patients (n = 1494) from 6 randomized, double-blind trials (N = 2543). Response was defined as a ≥30% reduction in Positive and Negative Syndrome Scale (PANSS) Total score by Week 8 of treatment. Analyzed predictors were change in individual PANSS items at Weeks 1 and 2. A decision tree was constructed using classification and regression tree (CART) analysis to identify predictors that most effectively differentiated responders from non-responders.

**Results:**

A 2-branch, 6-item decision tree was created, producing 3 distinct groups. First branch criterion was a 2-point score decrease in at least 2 of 5 PANSS positive items (Week 2). Second branch criterion was a 2-point score decrease in the PANSS excitement item (Week 2). "Likely responders" met the first branch criteria; "likely non-responders" did not meet first or second criterion; "not predictable" patients did not meet the first but did meet the second criterion. Using this approach, response to treatment could be predicted in most patients (92%) with high positive predictive value (79%) and high negative predictive value (75%). Predictive findings were confirmed through analysis of data from 2 independent trials.

**Conclusions:**

Using a data-driven approach, we identified decision rules using early change in the scores of selected PANSS items to accurately predict longer-term treatment response or non-response to atypical antipsychotic therapy. This could lead to development of a simple quantitative evaluation tool to help guide early treatment decisions.

**Trial Registration:**

This is a retrospective, non-intervention study in which pooled results from 6 previously published reports were analyzed; thus, clinical trial registration is not required.

## Background

Over 66% of patients with chronic schizophrenia who are started on atypical antipsychotics for treatment of moderate to severe symptoms will fail to show moderate improvement after 3 months of treatment, and 1 in 3 will fail to show even minimal improvement [1,2]. When treatment does not rapidly bring about symptomatic improvement, patients have less long-term functional progress, higher health care costs, and a reduced likelihood of remission [3]. These patients are more likely to discontinue treatment compared to those who experience rapid improvement [4], and treatment discontinuation can have devastating consequences in this population [5]. The ability to rapidly identify patients who are unlikely to improve on a given treatment allows for early intervention (eg, a change in dosage, a new schedule of delivery, an alternative medication) that could lead to better symptomatic relief, increased treatment adherence, decreased burden of suffering for patients and families, and decreased health care resource utilization.

Until recently, scientists and clinicians believed that at least 3 to 4 weeks of treatment were needed for patients to experience clinically meaningful symptomatic improvement in response to antipsychotic therapy. It has now been convincingly shown that a substantial amount of improvement occurs within 2 weeks or less of initiating treatment [6-8]. Failure to respond within this time frame has been shown in multiple post hoc analyses to strongly predict later non-response with continued use of the same agent [1,9,10]. More recently, the predictive power of early response/non-response was shown in a prospective study of patients with chronic disease [2] and in a retrospective study of patients experiencing first episode psychosis [11].

To date, studies assessing early symptom improvement [1,2,9,10] as a predictor of longer-term response have used a predetermined percent reduction from baseline in the Brief Psychiatric Rating Scale (BPRS) [12] Total score or the Positive and Negative Syndrome Scale (PANSS) [13] Total score at a point early in treatment as the criterion for differentiating likely responders and likely non-responders; for example, a 20% improvement in PANSS Total score at Week 2 of treatment might identify patients likely to respond at Week 8. However, the BPRS and PANSS are rarely used in clinical practice. The BPRS consists of 18 items and requires 20 to 30 minutes to administer, and the PANSS consists of 30 items and takes up to 40 minutes to administer. Time constraints related to patient care make using these scales to guide clinical decision making all but impossible.

In this study we used data from 6 randomized, double-blind clinical trials of atypical antipsychotic medications for treatment of moderately to severely ill patients with chronic schizophrenia to develop a simple decision tree employing early symptom improvement to predict longer-term response to treatment. Specifically, we used classification and regression tree (CART) analysis [14,15] to identify what *amount of change *in which of the *fewest PANSS symptom measures *at the *earliest time *in treatment is *most predictive *of response or non-response at Week 8 of treatment. Once a decision tree was created, we tested its validity by applying it to predict response in 2 independent studies with similar designs and patient populations.

## Methods

### Patients

Data from patients who met criteria for moderate to severe illness (n = 1494) were pooled from 6 randomized, double-blind trials of at least 8 weeks duration that compared atypical antipsychotics in the treatment of adult patients with chronic schizophrenia (N = 2543). In 5 of the studies, olanzapine was compared to another atypical antipsychotic: risperidone in 2 trials [16,17]; ziprasidone in 2 trials [18,19]; and quetiapine in 1 trial [20]. One of these studies included randomization to haloperidol treatment [16], but data from this arm were not included in this analysis since we were constructing a decision tree pertaining only to atypical antipsychotics. In the sixth study, 3 fixed doses of olanzapine were used to assess the dose-response relationship of standard and higher-dose olanzapine [21]. All studies assessed efficacy and safety outcomes, and they ranged in duration from 8 to 52 weeks. For consistency, all analyses were limited to data through 8 weeks of treatment, the duration of the shortest study. Also, to ensure that the final model accurately reflected the clinical course of patients with chronic illness who have positive symptoms and are moderately to severely ill at presentation, the analysis was limited to data from patients who had a PANSS Total score ≥75 and a score ≥4 on at least 2 of the 4 BPRS positive symptom items (conceptual disorganization; suspiciousness; hallucinatory behavior; and unusual thought content) (n = 1494).

All participants met Diagnostic and Statistical Manual of Mental Disorders, Fourth Edition, Text Revised (DSM-IV TR) criteria for schizophrenia, schizophreniform disorder, or schizoaffective disorder, with the majority of patients having been diagnosed with schizophrenia. The mean patient age was between 35 and 45 years, mean duration of illness was >10 years, and the group mean PANSS Total scores were ≥75. Patients in 3 of the studies [16,19,20] had lower PANSS Total scores (range: 85-86), while the other 3 studies [17-19] enrolled patients who were more acutely ill (baseline PANSS Total range: 95-102). Two studies had a special focus: the first enrolled patients with prominent depressive symptoms [19], and the other enrolled patients with prominent negative symptoms [20]. Patient populations were otherwise very similar across all studies.

Data from the 6 pooled studies is hereafter referred to as the "learning data set." The learning data set was used to create the decision tree, which was intended for future use in predicting response to treatment for similar patients.

Once the decision tree had been developed, it was validated using 2 independent studies. The first was a 12-week prospective trial to assess whether early symptom change in response to risperidone could predict subsequent clinical and functional outcomes in patients with schizophrenia [2]. The second was a 28-week, randomized, double-blind comparator trial of olanzapine versus aripiprazole for treatment of schizophrenia [22]. Patients in these 2 studies were similar to those in the learning data set. The majority of patients in these studies were aged late 30 s to early 40 s, had been ill with schizophrenia for >15 years, and were moderately to severely ill at baseline, with mean PANSS Total scores in the 90 to 95 range.

All study protocols were approved by the respective ethical review boards at participating study sites and were conducted in accordance with Good Clinical Practice and the Declaration of Helsinki guidelines. All patients or their legal guardians gave written informed consent prior to undergoing any study procedure or receiving any study treatment. The 6 pooled studies and 2 validation studies are summarized in Table 1. Detailed descriptions are available in their respective published reports [2,16-22].

**Table 1:** Characteristics of the 6 Studies Comprising the Learning Dataset and the 2 Studies Used for Validation of the CART-derived Decision Tree

								Discontinuation Rate, n (%)	
Study	Duration (weeks)	Design	Outcomes	Compounds	N	n (%)	Inclusion Criteria		
								Through Week 2	Through Week 8
**Learning Data Set Studies**									
Keefe[16]	52	Double-blind Randomized Controlled Flex-dose	Neurocognitive; Psychosocial; Efficacy; Safety	Olanzapine Risperidone	159 158	148 (47%)	Schizophrenia or schizoaffective disorder Inpatient and outpatient Age 18 to 65 Score ≥18 on the BPRS (ext) and ≥4 on ≥2 positive items of the PANSS	19 (11%) 16 (10%)	24 (14%) 32 (19%)
Tran[17]	28	Double-blind Randomized Controlled Flex-dose	Efficacy; Safety	Olanzapine Risperidone	172 167	227 (67%)	Schizophrenia, schizophreniform disorder, schizoaffective disorder Inpatient and outpatient Age 18 to 65 BPRS (ext) score ≥42	15 (9%) 21 (13%)	32 (20%) 29 (18%)
Breier[18]	28	Double-blind Randomized Controlled Flex-dose	Efficacy; Safety	Olanzapine Ziprasidone	277 271	122 (22%)	Schizophrenia Inpatient and outpatient Age 18 to 75 Scores ≥42 on the BPRS (ext), ≥4 on ≥1 positive symptom item of the PANSS, and CGI-S rating ≥4 (moderately ill)	27 (10%) 48 (18%)	39 (14%) 52 (19%)
Kinon[20]	24	Double-blind Randomized Controlled Flex-dose	Negative Symptoms; Functional Outcome; Efficacy; Safety	Olanzapine Quetiapine	171 175	142 (41%)	Schizophrenia, schizoaffective disorder Outpatient Age 18 to 65 Score ≥4 on ≥3, or ≥5 on ≥2 of the 7 negative symptom items of the PANSS, and ≤60 (moderate difficulties) on the GAF	13 (8%) 17 (10%)	39 (23%) 44 (25%)
Kinon[19]	24	Double-blind Randomized Controlled Fixed-dose	Depressive Symptoms; Efficacy; Safety	Olanzapine Ziprasidone	202 192	62 (16%)	Schizophrenia, schizoaffective disorder Inpatient and outpatient Age 18 to 60 Scores ≥16 (mild depression) on the MADRS and ≥4 (pervasive feelings of sadness or gloominess) on item 2 (reported sadness) of the MADRS	23 (11%) 31 (16%)	49 (24%) 63 (33%)
Kinon[21]	8	Randomized Double-blind Fixed-dose	Dose-response relationship; Efficacy; Safety	Olanzapine 10 mg 20 mg 40 mg	199 200 200	486 (81%)	Schizophrenia or schizoaffective disorder Suboptimal treatment on present antipsychotic, but not treatment resistant	83 (14%)	109 (18%)
**Validation Studies**									
Kinon[2]	12	Prospective Randomized Double-blind Flex-dose Parallel	Early improvement predictive of later response	Risperidone	628	438 (70%)	Schizophrenia, schizophreniform disorder, schizoaffective disorder BPRS Total ≥45; score of ≥4 (moderate) on at least two of the following BPRS items: conceptual disorganization, suspiciousness, hallucinatory behavior, and unusual thought content; CGI-S rating ≥4 (moderately ill) Exacerbation of illness within two weeks of Visit 1	3 (1%)	127 (24%)
Kane[22]	28	Double-blind Randomized Controlled Flex-dose	Efficacy Safety	Olanzapine Aripiprazole	281 285	415 (73%)	Schizophrenia Age 18 to 65 years Initial PANSS Total score ≥75; score of ≥4 on one of the PANSS positive, and score of ≥4 on the CGI-S; score of ≥3 on the CGI-I at Visit 2.	27 (10%) 39 (14%)	44 (16%) 48 (17%)

Abbreviations: BPRS (ext) = Brief Psychiatric Rating Scale extracted from the PANSS; CART = classification and regression tree; CGI-I = Clinical Global Impression-Improvement; CGI-S = Clinical Global Impression-Severity; GAF = Global Assessment of Functioning; MADRS = Montgomery-Asberg Depression Rating Scale; N = total number of patients within each treatment group for each study; n (%) = number and proportion of patients from each study who met illness severity criteria for inclusion in this analysis; PANSS = Positive and Negative Syndrome Scale.

### Assessments and Definitions

In studies from the learning data set and the 2 trials used to assess the validity of the final model, patients had been evaluated at baseline and at all subsequent visits (Weeks 1, 2, 3, 4, 6, and 8) using the PANSS_1-7_, a 30-item rating instrument of positive, negative, and general psychopathology symptoms (each item scored from 1 = absent to 7 = severe) and the Clinical Global Impression-Severity (CGI-S) scale [23], a clinician assessment of overall severity of illness (scored from 1 = normal, not at all ill to 7 = extremely ill). The Heinrich Quality of Life scale (QLS) [24], a 21-item, physician-rated scale designed to assess functional outcomes in patients with schizophrenia, was administered at Weeks 1 and 8. Each item of the QLS is rated on a 7-point scale (0-6), and total scores range from 0 to 126, with higher scores indicating less functional impairment.

Response was defined as a ≥30% reduction from baseline in PANSS_1-7 _Total score by Week 8 of treatment, a threshold chosen to reflect symptom improvement that was better than "minimal"[8], yet still realistic at this early time point. Patients with ≥30% improvement at Week 8 were identified as responders; those with <30% improvement at Week 8 were identified as non-responders. Missing data from each of the studies were handled by the method of last-observation-carried-forward (LOCF).

### Statistical Analysis

#### Construction of Decision Tree

We used CART analysis to determine which individual components of symptom improvement (input variables) were most predictive of longer-term response. In CART analysis, a set of rules is developed for dividing a large heterogeneous population into smaller, more homogeneous groups with respect to a particular target variable [25]. For this analysis, the target was response to treatment, as previously defined. The division or branching process was accomplished by searching all input variables (ie, potential predictors) and all values of each input variable to find the individual variable and its cut-off value that best classified patients as responders or non-responders, as determined by some statistical criteria. For each subgroup identified by the first branching step, the process was repeated, creating a tree structure known as a classification tree or decision tree. Input variables were absolute PANSS symptom scores at baseline and change from baseline in individual symptom scores at Weeks 1 and 2. Baseline characteristics such as gender, age, and illness duration were also included as input variables. Several input variables produced similar decision trees, and combining symptom scores that were highly correlated allowed us to form composite variables that had greater predictive power. Examples included "at least a 2-point drop in symptom 1 and symptom 2." The amount of change, the composition of symptoms, and the number of symptoms included in the composite variable were explored broadly. Once the final composite input variable was chosen, we limited branching of the resulting decision tree in an effort to create a simple tool that could be used in clinical practice. Consideration was given to how much additional accuracy a partition added to the entire tree to warrant the complexity that resulted from having added it.

#### Assessing Validity

To ensure that our results were not driven by any one study or compound, we applied the model to data from the learning data set stratified by study and by compound, and calculated the positive predictive value (PPV), negative predictive value (NPV), percent not predictable, and percentage of patients who were misidentified (predicted to be a responder, but did not respond, or predicted not to respond, but did respond) for each subgroup.

To ensure that our Week 8 threshold for response -- ≥30% improvement in PANSS_1-7 _Total score -- had not unduly affected the final tree, we repeated the analysis using thresholds for response of ≥20% and ≥40% improvement. Finally, we calculated PPV, NPV, and percentage not predictable using the same degree of change in PANSS items from the final model, but with timing of that change defined by week of treatment (Weeks 1, 2, 4, 6, and 8) to ensure that a reasonable time point to assess early improvement had been chosen.

To assess the degree to which the criteria identified at Week 2 could accurately predict response beyond Week 8, we applied the model to data from Weeks 12 and 24 that were pooled from the 5 studies in the learning dataset lasting ≥24 weeks.

To assess the external validity of the tree, we applied it to data from 2 independent studies with similar patient populations and calculated the resulting PPV, NPV, percentage not predictable, and percentage of patients misidentified.

#### Baseline and Visit-wise Assessment of Predicted Response Groups

Baseline characteristics of likely responder, likely non-responder, and not predictable groups were compared using analysis of variance (ANOVA) and the Cochran-Mantel-Haenszel test accounting for study effect. Visit-wise mean PANSS Total scores were compared by group using ANOVA adjusted for study at each time point. As a validation of results obtained using PANSS scores, we analyzed by group the distribution of CGI-S categories (normal to mild [1-3], moderate [4], and marked to extreme [5-7]) at each visit using Fisher's exact test. Changes in QLS score from baseline to Week 8 were compared between groups using ANOVA adjusted for study effect.

## Results

### Construction of Decision Tree

Exploratory analyses consistently showed that the key predictors of response were whether a patient had at least a 2-point drop in any 1 of the following 5 items from the PANSS Positive subscore: 1-Delusions; 2-Conceptual Disorganization; 3-Hallucinatory Behavior; 6-Suspiciousness; and 23-Unusual Thought Content. A single composite variable was identified as the optimal first partition splitting-variable: at least a 2-point drop in at least 2 of these items.

**Figure 1:**
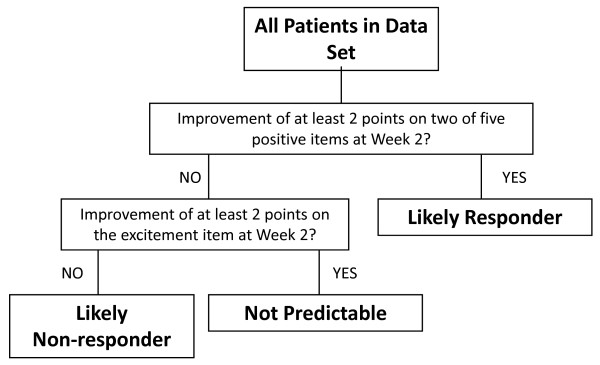
**Final CART-derived decision tree for early symptom change predicting later response**. Response is defined as ≥30% improvement from baseline in PANSS Total score at Week 8. Abbreviations: PANSS = Positive and Negative Syndrome Scale.

The final decision tree for predicting longer-term response to treatment based on early symptom improvement involved 2 branches and 3 predicted outcome groups: likely responders, likely non-responders, and not predictable (Figure 1). At the first level, patients were partitioned based on whether they had improved by at least 2 points on at least 2 items of the composite variable at Week 2. Patients who met this criterion were identified as likely responders. Patients who did not meet this criterion were further partitioned based on whether they had improved by at least 2 points on the excitement item (4-Excitement) at Week 2. Patients who met neither the first nor second level criteria were identified as likely non-responders; patients who did not meet the first branch criterion, but met the second branch criterion were identified as not predictable.

**Figure 2:**
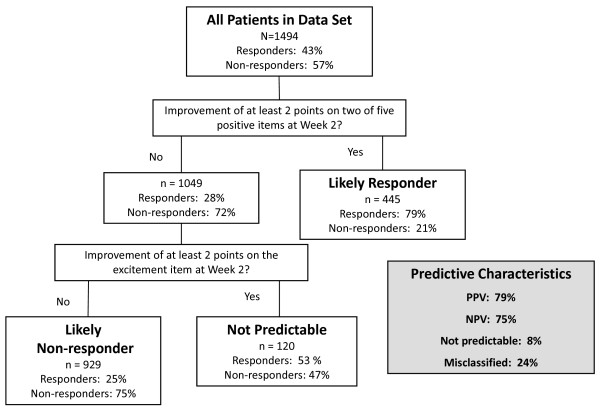
**Final CART-derived decision tree for early symptom change predicting later response, learning data set**. Response is defined as ≥30% improvement from baseline in PANSS Total score at Week 8. Abbreviations: PANSS = Positive and Negative Syndrome Scale.

The decision tree applied to the learning data set is illustrated in Figure 2. Of the 1494 patients in the learning data set, 644 (43%) ultimately demonstrated response, defined as a ≥30% improvement in PANSS Total score at Week 8. Using the first branch criterion, 445 patients were identified as likely responders, of which 352 actually responded (PPV = 79%). Of the 1049 patients who did not meet the first branch criterion, 755 ultimately did not respond (NPV = 72%). The NPV could be improved further by using the second branch criterion to separate the 1049 patients into likely non-responders and not predictable. Of the 929 patients who did not meet first and second branch criteria at Week 2, 698 did not respond (NPV = 75%). The number of patients in whom a prediction could not be made was small (120/1494 = 8%). Of the 24% (326/1374) of patients who were misidentified, 95 were non-responders who had been identified as likely responders, and 231 were responders who had been identified as likely non-responders.

Week 8 response was predicted more accurately by improvement in individual PANSS items at Week 2 than by improvement at Week 1 or by baseline characteristics. We also investigated the predictive value of the CGIS score at Week 2 as a single variable for predicting later response. A 2-point drop in CGI-S at Week 2 resulted in a PPV of 83% and an NPV of only 56%. Since only a small proportion of patients (5.7%) had this level of improvement by Week 2, we also calculated the predictive characteristics of a 1-point drop in CGI-I score, a level of improvement achieved by 49% of patients. The results -- a PPV of 67% and a NPV of 60% -- indicated that this degree of change in this variable was a far less reliable predictor of later response than change in a cluster of PANSS Positive symptoms at that same point in time.

### Assessing Validity

With regard to differences across studies (Table 2), the range of PPVs (46% to 85%) and NPVs (60% to 95%) was fairly wide, reflecting the variation in results known to exist between trials of antipsychotic agents. However the PPVs and NPVs were distributed evenly across their respective ranges, and there was no single outlier that pulled results in one direction or the other. Concerning consistency across compounds (Table 3), PPVs were consistently high (66% to 82%), except for the PPV seen in the study in which quetiapine was the comparator (40%). We believe that this difference may be explained by the slower rate of response seen for patients in this trial. In this trial, recruited patients had prominent negative symptoms, a clinical characteristic associated with poor response to antipsychotics. Also, this trial employed a slower rate of titration than was used in the other studies. Even with these differences, the ratios of patients meeting response criteria at Week 2 to patients meeting criteria at Week 8, that is, the post-test to pre-test ratios, were very similar between the quetiapine study (40%/20% = 2) and the pooled studies (79%/43% = 1.8), suggesting that the model developed in this analysis was consistent, regardless of the rate of response at Week 2. NPVs by compound (range 73% to 88%) fell within a 15-point range, with no outliers present. A slow rate of response would have had greater impact on PPV than on NPV.

**Table 2:** CART-derived Decision Tree Outcomes for the Learning Data Set and its Component Studies

	**Positive** **Predictive** **Value**	**Negative** **Predictive** **Value**	**Not** **Predictable**	**Misidentified**
	
Learning Data Set	79%	75%	8%	24%
Keefe[16] (n = 148)	46%	95%	5%	14%
Tran[17] (n = 227)	76%	60%	9%	35%
Breier[18] (n = 122)	85%	68%	12%	25%
Kinon[19] (n = 142)	63%	81%	8%	24%
Kinon[20] (n = 52)	58%	89%	5%	16%
Kinon[21] (n = 486)	84%	72%	6%	23%

Abbreviations: CART = classification and regression tree.

**Table 3:** CART-derived Outcomes for the Learning Data Set and its Component Compounds

	**Positive** **Predictive** **Value**	**Negative** **Predictive** **Value**	**Not** **Predictable**	**Misidentified**
	
Learning Data Set (n = 1494)	79%	75%	8%	24%
Olanzapine (n = 1005)	81%	73%	8%	26%
Quetiapine (n = 71)	40%	88%	6%	19%
Risperidone (n = 185)	66%	77%	7%	26%
Ziprasidone (n = 233)	82%	78%	9%	21%

Abbreviations: CART = classification and regression tree.

A sensitivity analysis using ≥20% and >40% thresholds for response at Week 8 resulted in similar trees produced using a >30% threshold, except that only a 1-point improvement in each of the identified PANSS items was needed when the lower threshold was used.

Of the 1008 patients pooled from the 5 studies lasting ≥24 weeks, 41% and 43% of patients demonstrated a 40% reduction in PANSS Total score at Weeks 12 and 24, respectively. When model criteria were used to predict response at Weeks 12 and 24, the PPVs were 71% and 51%, respectively and the NPVs were 86% and 82%, respectively. These results were only slightly less robust than those observed with the learning dataset (PPV = 79% and NPV = 75%).

The percentage of patients who demonstrated a ≥30% response by Week 8 in the 2 independent validation studies was 37.1% for Kinon et al. [2] and 46.7% for Kane et al. [22], which were similar to that of patients who achieved this degree of response in the pooled studies (also 42%). The predictive accuracy of the decision tree applied to each of the independent studies was highly consistent with that seen for the learning data set (PPV = 70% and 77%, respectively; NPV = 77% and 68%, respectively) (Table 4).

**Table 4:** CART-derived Decision Tree Outcomes for the Learning Data Set and Validation Studies

	**Positive** **Predictive** **Value**	**Negative** **Predictive** **Value**	**Not** **Predictable**	**Misidentified**
	
Learning Data Set	79%	75%	8%	24%
Kinon[2]	70%	77%	7%	25%
Kane[22]	77%	68%	7%	29%

Abbreviations: CART = classification and regression tree.

**Figure 3:**
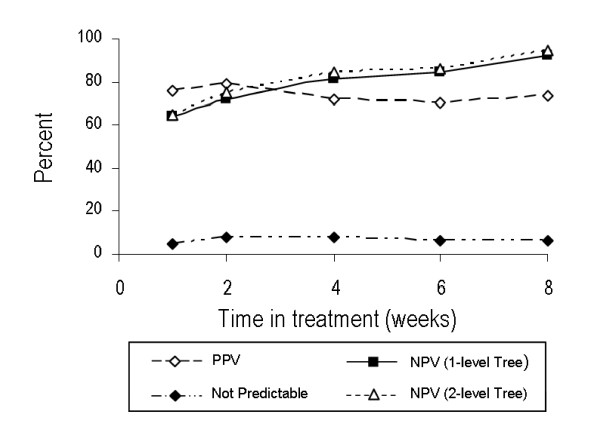
**PPV and NPV using one- and two-branch decision tree models**. Percentage of patients identified as not predictable as determined using decision tree rules, but with response defined by week of treatment. Abbreviations: NVP = negative predictive value; PPV = positive predictive value.

Positive predictive value and NPV using the described 1-branch and 2-branch decision tree models, and the percentage of patients in the learning data set who were not predictable are shown by week of treatment in Figure 3. Although the range of PPV throughout the study was narrow, it peaked at Week 2 (79%), after which it fell to a low of 71% at Week 6. In contrast, regardless of whether the 1-branch or 2-branch decision tree was used, NPV gradually increased throughout the study. Using the 2-branch model, NPV was 65%, 75%, 85%, and 86% for Weeks 1, 2, 4, and 6, respectively.

#### Baseline and Visit-wise Assessment of Predicted Response Groups

Baseline characteristics and illness severity for likely responder, likely non-responder, and not predictable groups are shown in Table 5. A significantly greater proportion of patients identified as likely responders or not predictable were female compared to patients identified as likely non-responders (p = .02). Compared to likely non-responders, these 2 groups were also more likely to have had higher illness severity as measured by PANSS Total (p < .001), PANSS Positive (p < .001), PANSS Negative (p = .002), and CGI-S (p < .001) scores at baseline.

**Table 5:** Baseline Demographics and Illness Severity for Predicted Outcome Groups

	**Likely Responders**	**Likely Non-responders**	**Not Predictable**	**p-value**^**a**^
	
n (%)	445/1494 (29.8)	929/1494 (62.2)	120/1494 (8.0)	
Age in years, mean (SD)	39.1 (11.1)	40.4 (10.6)	38.9 (10.4)	.06
Female gender, n (%)	167 (37.5)	295 (31.8)	51 (42.5)	.02
Ethnicity/Race, n (%)				
White	219 (49.2)	474 (51.0)	56 (46.7)	.53
Black	141 (31.7)	309 (33.3)	43 (35.8)	
Hispanic	85 (19.1)	146 (15.7)	21 (17.5)	
Diagnosis, n (%)				
Schizophrenia	345 (77.5)	743 (80.0)	101 (84.2)	.26
Schizoaffective disorder	95 (21.3)	186 (20.0)	18 (15.0)	.31
Schizophreniform disorder	5 (1.1)	0 (0.0)	1 (0.8)	.003
Age at illness onset in years, mean (SD)	23.8 (8.8)	23.4 (8.3)	23.0 (7.4)	.53
Duration of illness in years, mean (SD)	15.3 (10.9)	17.0 (10.7)	15.9 (10.5)	.02
Number of prior episodes, median	5	6	5	
PANSS Total score, mean (SD)	101.5 (16.1)	95.4 (14.0)	101.9 (17.3)	<.001
PANSS Positive score, mean (SD)	25.6 (5.1)	23.5 (4.2)	26.1 (4.7)	<.001
PANSS Negative score, mean (SD)	26.0 (5.9)	24.9 (5.5)	25.4 (6.0)	.002
CGI-S score, mean (SD)	4.8 (0.8)	4.7 (0.7)	4.8 (0.7)	<.001
Weight in kg, mean (SD)	83.0 (22.9)	84.9 (20.5)	82.6 (22.0)	.24

Abbreviations: CGI-S = Clinical Global Impression-Severity^23^; n = number in group; PANSS = Positive and Negative Syndrome Scale^13^; SD = standard deviation.

^a^Comparisons based on analysis of variance (ANOVA) and the Cochran-Mantel-Haenszel test accounting for study effect.

**Figure 4:**
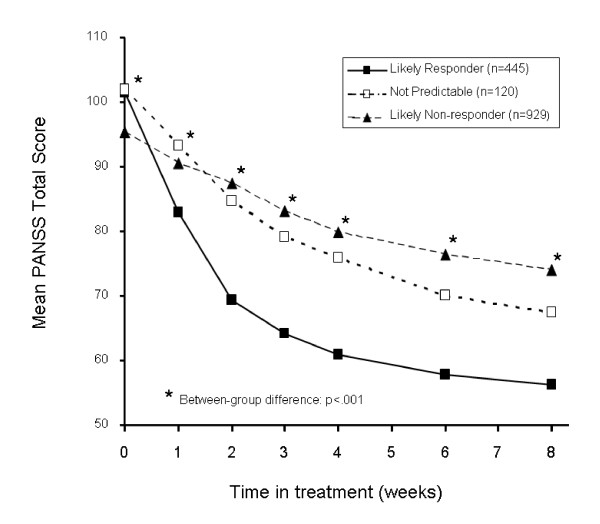
**Visit-wise mean PANSS Total scores**. Percentage of patients in the learning data set identified as likely responders, likely non-responders, and not predictable using decision tree rules. Abbreviations: PANSS = Positive and Negative Syndrome Scale.

**Figure 5:**
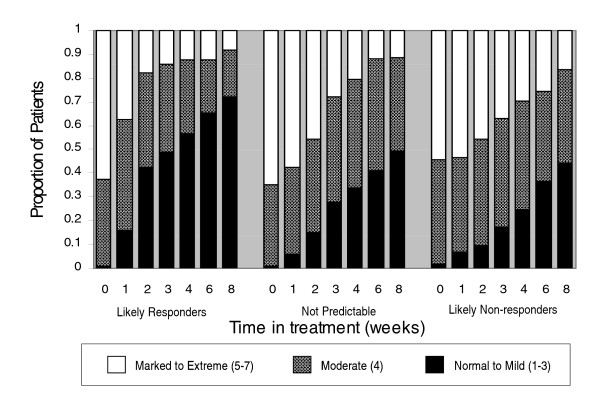
**CGI-S scores by illness severity over time**. Percentage of patients by CGI-S scores (normal to mild [1-3], moderate [4], and marked to extreme [5-7]) is shown over time by outcome group. Abbreviations: CGI-S = Clinical Global Impression-Severity.

Visit-wise mean PANSS Total score for patients in the learning data set are shown in Figure 4. As had been seen in previous response prediction studies [2,11], the reduction in mean PANSS Total score was significantly lower (p < .001) at every post-baseline visit for likely responders compared to likely non-responders. A separation between group means was evident as early as Week 1 and this gap widened by Week 2, at which time likely responders had a 32-point improvement, 4 times that seen in likely non-responders. The profile of mean PANSS score over time for the not predictable group lay between those of the other 2 groups. The percentage of patients with CGI-S scores indicating normal to mild (1-3), moderate (4), and marked to extreme (5-7) illness severity is shown over time by outcome group in Figure 5. By study end, 72% of likely responders had normal to mild symptoms compared to 49% in the not predictable group and 45% in the likely non-responder group (p < .001). Mean improvement from baseline to Week 8 in QLS Total scores was 13.8, 7.1, and 3.7 points for the likely responder, not predictable, and likely non-responder groups, respectively (p < .001).

## Discussion

We used CART analysis on data pooled from 6 large, double-blind, randomized atypical antipsychotic therapy trials to develop a simple 2-branch decision tree that uses the amount of improvement in 6 PANSS items after 2 weeks of treatment to predict the likelihood of longer-term response to continued use of the same treatment by Week 8. When the model was applied to the data set from which it was created, we identified likely responders with 79% accuracy, likely non-responders with 75% accuracy, and a small group of patients (8%) in whom a prediction could not be made. In analyses of the 3 predicted response groups, patients identified as likely responders were more commonly female and had a shorter duration of illness than likely non-responders. They were also more severely ill at baseline across a spectrum of symptom domains, and showed significantly more rapid and more pronounced improvement over time.

At the first branch of the decision tree, patients were assessed for improvement in a composite variable consisting of 5 of the PANSS Positive items. A separate analysis of individual PANSS items showed strong correlation between these 5 items, lending strength to our model. Several other prediction studies have identified early improvement in positive symptoms as the primary predictor of later response and non-response. Correll et al [9] evaluated BPRS scores as a predictor of later non-response and identified lack of improvement in the thought disturbance factor as the strongest predictor of non-response. Similarly, in 2 studies in which early improvement in PANSS Total score was shown to be predictive of later response [1,2], 40% of the overall improvement was due to the PANSS Positive subscore, with the other subscores contributing only 27% to 33% of the improvement.

Finding that early improvement in positive symptoms accurately predicted later improvement confirmed results of earlier studies and extended these results by specifying how early improvement can be assessed and how much improvement is required to accurately predict later response. We focused on a population of patients with chronic schizophrenia who were moderately to severely ill, with predominantly positive symptoms. This describes the majority of patients who come to medical attention and who are initiated or re-initiated on antipsychotic treatment, or who have their current medication changed or adjusted. Also, antipsychotics are most effective in treating positive symptoms, so a model that predicts later response based on early response is likely to include them. Whether this model applies to patients without prominent positive symptoms will be an interesting area for future research.

Our decision tree was strengthened and stabilized by the large number of patients and the diversity of studies pooled to create our data set. Although each of the studies had a similar design and similar enrollment criteria, they were carried out at both U.S. and international sites, included 5 different atypical antipsychotics, and were completed over an 11-year period. An analysis of model performance by study and by compound suggested that the creation of the model had not been driven by any one study or any one compound, but rather was a reasonable reflection of its component parts.

Many strategic decisions are made during a CART analysis, and the final usefulness of a classification tree depends heavily on the skill and judgment of the statisticians, scientists, and clinicians who determine the input variables and prune the output. We chose changes in PANSS item scores obtained at Weeks 1 and 2 as input variables based on prior work with prediction models. Our analysis of PPV and NPV by study week illustrates the trade-offs inherent in choosing the time for early assessment. While PPV stayed relatively stable regardless of the time point chosen, NPV improved as treatment time passed. However, in the clinical setting, delaying a treatment decision in order to improve NPV translates into additional suffering for some patients who will not improve on present therapy, so we included only very early time points as input variables. We also chose individual PANSS items as input variables rather than the PANSS Total score. The practicality of using a tool consisting of only 6 questions rather than administering the entire 30-question PANSS cannot be overemphasized.

Pruning is another area where investigator input is required. A decision tree that is too complex will likely reflect the idiosyncrasies of the data set used to create it, while a tree that is too simple may not fit any data set very well. In constructing this decision tree, we considered pruning back to the first branch, at which point the NPV was 72%. By including the additional branch, we improved the NPV to 75%, but in doing so, added another level of complexity to what could have been a single-question algorithm. This balance between complexity and increased predictive value must be weighed by the practicing clinician.

We found that our model was more accurate in predicting response at Week 8 based on response at Week 2 than change in the CGI-I at Week 2. Accurate prediction of later response may require a careful assessment of the current severity of a few distinct symptoms rather than a summary measure of change over 2 weeks that includes consideration of the patient's history, psychosocial circumstances, symptoms, behavior, and functioning.

Constructing a decision tree using CART analysis requires having access to a very large database with a consistent set of patients (eg, similar inclusion/exclusion criteria), consistent study design (eg, timing, blinding, randomization), and consistent clinical measurements (eg, PANSS). With the appropriate database, CART analysis is a powerful method for answering clinical questions. It is non-parametric; that is, no assumptions are made regarding the underlying distribution of variables, and it permits consideration of both continuous and categorical data. It can handle data sets with large numbers of input variables, each with many possible values; identify significant high-order interactions among variables; detect and quantify non-linear relations; determine which predictors can be dropped because they contain redundant information; and handle missing values through a process of "surrogate substitution." The tree can be simplified because the response variables are, for the most part, conditionally independent of variables not in the tree. CART analysis is limited in that it can be unstable; that is, a small change in the input variables or a fresh data sample can lead to construction of a very different classification tree. In addition, CART analysis is limited in that it can account for only one response variable [14,15].

The answer provided by CART analysis (the percent likelihood of response) is in line with the mindset of most clinicians. After initiating treatment, clinicians routinely evaluate their patients, make judgments about their progress, and decide whether they should continue treatment or make a change. This decision tree simply provides evidence-based tools, an algorithm, and associated PPV and NPV, to help quantify the decision-making process. Clinicians must still actively consider the unique presentation of each patient; for example, a patient with a high probability of failure as determined by the algorithm may benefit from continuing the treatment if they have already failed several previous medications and a patient with a high probability of success may have treatment discontinued due to an intolerable adverse event.

We recognize that when outcomes have been categorized as dichotomous (response/non-response), other approaches such as logistic regression can be used to create a predictive model. The strengths of CART compared with logistic regression are worth noting in this setting. First, CART analysis offers a great deal of flexibility because it requires very few assumptions about the data. Due to its natural branching process, CART is able to handle many dozens of potential predictor variables as well as potential interactions between predictors and non-linear effects of individual predictors, and missing data. CART also has algorithms that account for missing data amongst the predictors; whereas with logistic regression, entire observations are eliminated if a single predictor is missing. Furthermore, at the end of a CART analysis, subgroups (responders and non-responders) are specifically defined due to the variable selection and cut-offs created by the methodology. With logistic regression, significant predictors are identified, but the coefficients do not automatically define subgroups of patients. Finally, our experience suggests that the stepwise, dichotomous approach that is used with a decision tree more closely mimics clinical decision-making than use of odds ratios which indicate association, but do not clearly direct treatment decisions.

Two patient populations identified in our analysis deserve special attention from a clinical standpoint: first, the 8% of patients in whom a prediction could not be made and second, the 24% who were misidentified. The decision criteria laid out in this model allow for a category of patients (those who show improvement in the excitement item at Week 2, but who do not show significant improvement in positive symptoms) in whom the prediction of later response is no better than a coin toss. Fortunately, this population is relatively small, <10% in 14 of the 15 analysis subsets (learning data set analysis, validation analyses, analysis by study, and analysis by compound). Of those patients in the learning data set who were misidentified, the majority was identified as non-responders, but eventually did respond. The clinical ramifications of changing treatment when no change is needed versus continuing use of an ineffective treatment must be carefully weighed by all those involved.

Despite having developed this model using response data from Week 8, the strength of the model was diminished only slightly when used to predict response at Weeks 12 and 24. Further research is needed to see if a model derived using response data following longer-term treatment data might have greater accuracy in predicting response beyond Week 8.

Currently, this decision tree is appropriately used only for adult patients with chronic schizophrenia who are moderately to severely ill. It needs to be tested in other patient populations, such as those with chronic schizophrenia who are only mildly ill, and in patients with first episode psychosis. In addition, it needs to be validated in a prospective study design and to be assessed for inter-rater reliability. It is possible that introduction of additional variables, functional measurements or patients' perception of benefit, for example, would have altered the final decision tree. Further model building using data from different populations and different input variables is needed to establish the stability of this particular decision tree and to perhaps construct a tree with even better predictive characteristics and user-friendliness. In addition, were a tool such as this decision tree to be developed for use in the clinical setting, input on clear and effective phrasing would be needed from clinicians. For example, would verbal descriptions such as "substantial improvement" or numeric descriptions such as "a 2-point drop" be most preferred?

## Conclusions

We used CART analysis of a large pooled data set to construct a simple decision tree that was found to be predictive of later response/non-response based on improvement in 6 individual PANSS items over 2 weeks of treatment. These findings could serve as a foundation for development of a clinically relevant evaluation tool for guiding treatment decisions early in the course of atypical antipsychotic drug therapy.

## Competing interests

All authors are employees of Eli Lilly and Company and/or one of its subsidiaries, and all authors own stock in Eli Lilly and Company.

## Authors' contributions

Authors SR and LC conceived of and designed this study. Author LC performed the statistical analysis. Author SR helped to draft the manuscript. All authors substantially contributed to interpretation of the data and critically revised the manuscript, and read and approved the final manuscript.

## Pre-publication history

The pre-publication history for this paper can be accessed here:

http://www.biomedcentral.com/1471-244X/11/23/prepub
